# Autoimmune Disease Associated *CLEC16A* Variants Convey Risk of Parkinson’s Disease in Han Chinese

**DOI:** 10.3389/fgene.2022.856493

**Published:** 2022-03-30

**Authors:** Hui-Hui Fan, Lei Cui, Xiao-Xia Jiang, Ya-Dan Song, Shu-Shu Liu, Ke-Yun Wu, Hao-Jia Dong, Miao Mao, Begench Ovlyakulov, Hong-Mei Wu, Jian-Hong Zhu, Xiong Zhang

**Affiliations:** ^1^ Department of Preventive Medicine, Institute of Nutrition and Diseases, Wenzhou Medical University, Wenzhou, China; ^2^ Department of Neurology, Institute of Geriatric Neurology, the Second Affiliated Hospital and Yuying Children’s Hospital, Wenzhou Medical University, Wenzhou, China

**Keywords:** CLEC16A, Parkinson’s disease, autoimmune diseases, genetic variation, association

## Abstract

CLEC16A is a membrane-associated endosomal protein implicated in regulating autophagy and antigen presentation. Its genetic variants are broadly associated with multiple autoimmune diseases. Parkinson’s disease (PD), which undergoes autophagy disruption and neuroinflammation, has been clinically observed, for an extensive amount of time, to be associated with autoimmune diseases. In this study, we aimed to understand whether the autoimmune disease associated *CLEC16A* variants pleiotropically modulate PD risk. Five of such *CLEC16A* variants, including rs6498169, rs12708716, rs12917716, rs7200786, and rs2903692, were selected and analyzed in a Han Chinese cohort comprising 515 sporadic PD patients and 504 controls. Results showed that rs6498169 and rs7200786 were significantly associated with PD susceptibility (*p* = 0.005 and 0.004, respectively; recessive model, *p* = 0.002 and 0.001, respectively). Rs6498169 was also associated with the PD subtype of postural instability/gait difficulty (*p* = 0.002). Haplotype analysis showed that the AAG module in order of rs6498169, rs12708716, and rs2903692 was associated with the highest risk for PD (*p* = 0.0047, OR = 1.42, 95% CI = 1.11–1.82). Functional annotation analyses suggested that rs6498169 had high probability to affect transcription factor binding and target gene expression. In summary, the current study demonstrates that the autoimmune disease associated *CLEC16A* variants convey risk of PD in Han Chinese. Our findings suggest a pleiotropic role of *CLEC16A* and strengthen the link between PD and autoimmune diseases.

## Introduction

*CLEC16A* (C-type lectin domain family 16, member A; Chr16; Gene ID: 23274; MIM: 611303), encoding a large protein of 1,053 amino acids with a putative C-type lectin-like domain, is a membrane-associated endosomal protein (Soleimanpour et al., 2014). *CLEC16A* genetic variations have been associated with multiple autoimmune diseases including multiple sclerosis, type 1 diabetes, Crohn’s disease, Addison’s disease, rheumatoid arthritis, and juvenile idiopathic arthritis (Vitale et al., 2002; Hakonarson et al., 2007; International Multiple Sclerosis Genetics et al., 2007; Marquez et al., 2009; Martinez et al., 2010; Skinningsrud et al., 2010; Nischwitz et al., 2011; International Multiple Sclerosis Genetics et al., 2013). The broad association with autoimmune disorders suggests that *CLEC16A* may functionally link to autoimmunity by certain common pathogenic pathways. Indeed, CLEC16A has been implicated in regulating autophagy/mitophagy and antigen presentation. CLEC16A interacts with E3 ubiquitin ligase NRDP1 (neuregulin receptor degradation protein 1), controlling the volume of Parkin and its mastered mitophagy and thereby mediates murine β cell function and diabetogenesis (Soleimanpour et al., 2014). In addition, *Clec16a* knockdown in the nonobese diabetic mice protects against autoimmunity by altering the T cell selection, possibly through the inhibition of thymic epithelial cell autophagy (Schuster et al., 2015). *Clec16a* mutant mice generated by gene-trap insertion develop neurodegeneration featured by impaired motor behaviors, loss of Purkinje cells, and abnormal autophagy homeostasis (Redmann et al., 2016). CLEC16A also plays a role in multiple sclerosis *via* controlling the late endosome biogenesis-mediated human leukocyte antigen class II antigen presentation (van Luijn et al., 2015).

Parkinson’s disease (PD) is the second most common neurodegenerative disorder characterized by the loss of dopaminergic neurons in the substantia nigra pars compacta. The majority of PD is sporadic and its etiology is complex and remains largely unknown. Autophagy dysfunction is one of the key mechanisms involved in PD, leading to the abnormal degradation and aggregation of proteins such as α-synuclein (Michel et al., 2016). Mutations in *PRKN* (Parkin encoding gene) are the common cause of the early-onset PD (Kalia and Lang, 2015). Recent studies have also revealed the presence of α-synuclein-reactive T cells in PD patients and an association of its reactivity with preclinical and early PD, indicating a link of PD to autoimmunity (Sulzer et al., 2017; Lindestam Arlehamn et al., 2020).

Indeed, PD risk has been observed, for an extensive amount of time, as associated with autoimmune diseases. For instance, a Swedish epidemiological study showed that subsequent risks of PD are increased in patients with 6 autoimmune diseases, including amyotrophic lateral sclerosis, Graves’s disease/hyperthyroidism, Hashimoto’s disease/hypothyroidism, multiple sclerosis, pernicious anemia, and polymyalgia rheumatica (Li et al., 2012), whereas being a casual or causal association between PD risk and multiple sclerosis remains in debate (Pedemonte et al., 2013; Nielsen et al., 2014). Nonetheless, it is hypothesized that there are common genetic risk variants between PD and autoimmune diseases (Witoelar et al., 2017). Given the functional overlap of *CLEC16A* with PD pathogenic mechanisms and its broad association with autoimmune diseases, we aimed, in this study, to investigate whether the autoimmune disease associated *CLEC16A* variants convey risk of PD in a Han Chinese population.

## Materials and Methods

### Subjects

A total of 1,019 subjects of Han Chinese ethnicity from eastern China were recruited in this study, comprising 515 sporadic PD patients (263 males and 252 females) and 504 controls (265 males and 239 females). The median age of the patients and controls was 66 (interquartile range: 60–73) and 66 (interquartile range: 57–72) years old, respectively. All PD patients were diagnosed by two movement disorder neurologists, according to the UK Parkinson’s disease Society Brain Bank Criteria (Hughes et al., 1992). Patients with a family history of PD or with secondary and atypical parkinsonism were excluded. The controls had no neurological disorders according to medical history, physical, and laboratory examinations. All subjects participating in the study signed written informed consents. The study was performed under the approval No. LCKY 2020-66 by the Ethics Committee of The Second Affiliated Hospital and Yuying Children’s Hospital, Wenzhou Medical University.

### Selection of *CLEC16A* Risk Variants

Five autoimmune disease-associated risk variants of *CLEC16A* were selected, including rs6498169, rs12708716, rs7200786, rs2903692 and rs12917716. Among these, rs6498169, rs12708716 and rs7200786 were identified as susceptibility markers for multiple sclerosis (Vitale et al., 2002; International Multiple Sclerosis Genetics et al., 2007; International Multiple Sclerosis Genetics et al., 2013); rs2903692 and rs12917716 were found to be associated with type 1 diabetes (Hakonarson et al., 2007; Skinningsrud et al., 2010); rs6498169 was additionally associated with juvenile idiopathic arthritis and rheumatoid arthritis (Martinez et al., 2010; Skinningsrud et al., 2010); rs2903692 was additionally associated with Crohn’s disease and multiple sclerosis (Marquez et al., 2009; Martinez et al., 2010); rs12917716 was additionally associated with Addison’s disease, multiple sclerosis, and primary adrenal insufficiency (Skinningsrud et al., 2010; Nischwitz et al., 2011).

### Genotyping

Genomic DNA was extracted from peripheral blood using TIANamp Genomic DNA kit (Tiangen, Beijing, China) according to the manufacturer’s instruction. Four SNPs, including rs6498169, rs12708716, rs12917716, and rs7200786, were genotyped using SNaPshot at BGI Technology (Wuhan, China) as described previously (Zou et al., 2018). Rs2903692 was not successfully genotyped by SNaPshot, and therefore was genotyped by conventional PCR and sequencing. The PCR condition was initial denaturation at 95°C for 3 min, followed by 32 cycles of 95°C for 30 s, 60°C for 30 s, and 72°C for 60 s, and a final extension at 72°C for 5 min. The PCR products were sequenced at BGI Technology (Wuhan, China). All relevant primers were listed in Supplementary Table S1.

### Statistical Analysis

Statistical analyses were carried out using the Statistical Package for Social Science program (SPSS for Windows, version 23.0). Hardy-Weinberg equilibrium in genotype distribution was assessed using χ2 test. Following Kolmogorov-Smirnov test for normality, Mann-Whitney U test was used to evaluate age difference. The χ2 test was also used to assess differences in gender, genotype, allele, and haplotype frequencies between the PD patients and controls. The haplotype construction and association analysis were performed using SNPStats Online Version (https://www.snpstats.net/start.htm) (Sole et al., 2006). A backward elimination method as reported previously was used to identify the highest-risk haplotype for PD (Francis et al., 2007). Statistical power was calculated by the QUANTO version 1.2.4. A *p* value < 0.05 was considered statistically significant unless otherwise indicated.

### Function Annotation

Function annotations of the variants were obtained from HaploReg v4.1 (http://pubs.broadinstitute.org/mammals/haploreg/haploreg.php) and RegulomeDB (http://www.regulomedb.org/). HaploReg was used to annotate potential causal links to disease pathogenesis (Ward and Kellis, 2016). RegulomeDB was used to annotate known and predicted genetic variations in regulatory elements in intergenic regions of the human genome. A score ranging from 1 to 6 was provided to indicate the potential function and the lower score indicates a higher probability that a variant affects binding and gene expression (Boyle et al., 2012). The expression quantitative trait locus (eQTL) and splicing quantitative trait locus (sQTL) of the variants were analyzed by the GTEx Portal (https://gtexportal.org/).

## Results

### Association of the CLEC16A Variants with PD Susceptibility

Genotype distributions of the 5 variants in controls were in accordance with Hardy-Weinberg equilibrium (*p* > 0.05). The PD cases and controls were comparable in both gender and age (*p* > 0.05). Difference was considered only after Bonferroni correction (threshold for significance = 0.01). Results showed that significant difference between PD cases and control cases was found only in genotypes of rs6498169 and rs7200786 (*p* = 0.005 and *p* = 0.004, respectively; Table 1). No difference was found within rs12708716, rs12917716, and rs2903692.

**TABLE 1:** Genotype and allele frequencies of five *CLEC16A* variants in PD patients and controls.

Variant	Genotype, n (%)			*P*	Allele, n (%)		*P*	OR (95%CI)
rs6498169	GG	GA	AA	0.005^a^	G	A	0.042	1.199 (1.007–1.427)
Control	151 (30.0)	264 (52.4)	89 (17.7)		566 (56.2)	442 (43.8)		
PD	150 (29.1)	232 (45.0)	133 (25.8)		532 (51.7)	498 (48.3)		
rs12708716	AA	AG	GG	0.430	A	G	0.288	1.116 (0.912–1.366)
Control	293 (58.1)	186 (36.9)	25 (5.0)		772 (76.6)	236 (23.4)		
PD	288 (55.9)	192 (37.3)	35 (6.8)		768 (74.6)	262 (25.4)		
rs12917716	GG	GC	CC	0.055	G	C	0.026	1.221 (1.024–1.456)
Control	173 (34.3)	258 (51.2)	73 (14.5)		604 (59.9)	404 (40.1)		
PD	154 (29.9)	259 (50.3)	102 (19.8)		567 (55.0)	463 (45.0)		
rs7200786	AA	AG	GG	0.004^a^	A	G	0.044	1.207 (1.005–1.448)
Control	220 (43.7)	239 (47.4)	45 (8.9)		679 (67.4)	329 (32.6)		
PD	216 (41.9)	218 (42.3)	81 (15.7)		650 (63.1)	380 (36.9)		
rs2903692	GG	GA	AA	0.731	G	A	0.732	1.036 (0.846–1.268)
Control	289 (57.3)	188 (37.3)	27 (5.4)		766 (76.0)	242 (24.0)		
PD	286 (55.5)	204 (39.6)	25 (4.9)		776 (75.3)	254 (24.7)		

a *P* < 0.01. CI, confidence interval; OR, odds ratio; PD, Parkinson’s disease.

We further analyzed the rs6498169 and rs7200786 by three genetic models (additive, dominant, and recessive). Both SNPs were significantly associated with PD in the recessive model (*p* = 0.002, OR = 1.629, 95% CI = 1.203–2.205 for rs6498169; *p* = 0.001, OR = 1.895, 95% CI = 1.285–2.795 for rs7200786). The risk genotypes for PD were the AA of rs6498169 and the GG of rs7200786 (Table 2). The statistical power was 92% and 96%, respectively, for the recessive model of rs6498169 and rs7200786.

**TABLE 2:** Genetic model analysis of rs6498169 and rs7200786.

Model	Genotype	*P*	OR (95% CI)
rs6498169			
Recessive	GG + GA *vs.* AA	0.002^a^	1.629 (1.203–2.205)
Dominant	GG *vs.* GA + AA	0.728	1.049 (0.801–1.374)
Additive	GG *vs.* GA *vs.* AA	0.039	1.200 (1.009–1.428)
rs7200786			
Recessive	AA + AG *vs.* GG	0.001^a^	1.895 (1.285–2.795)
Dominant	AA *vs.* AG + GG	0.569	1.075 (0.838–1.379)
Additive	AA *vs.* AG *vs.* GG	0.046	1.205 (1.004–1.446)

a *P* < 0.01. CI, confidence interval; OR, odds ratio; PD, Parkinson’s disease.

We also extracted a total of 265 PD patients recorded with PD subtypes to understand their association with the five *CLEC16A* SNPs. The subtypes were classified into postural instability/gait difficulty (PIGD), tremor dominant, and indeterminate. Results showed that the genotype distribution of rs6498169 was significantly different (*p* = 0.002) between the PIGD patients and the controls. No other difference was found in the variants between the PD subtypes and the controls (Supplementary Table S2).

### Haplotype Analyses of the CLEC16A Variants

We analyzed whether haplotypes of these *CLEC16A* variants were associated with PD. Haplotypes were constructed in the following order: rs6498169, rs12708716, rs12917716, rs7200786, and rs2903692. As shown in Table 3, five haplotypes were listed as those with a frequency < 3.0% in both PD patients and controls were excluded. A significant difference (*p* = 0.034) in overall haplotype distribution was observed between the PD patients and controls. Two haplotypes, AAGAG (*p* = 0.02, OR = 1.47, 95% CI = 1.06–2.04) and AACGG (*p* = 0.03, OR = 1.47, 95% CI = 1.04–2.07), showed a significant difference between the cases and controls.

**TABLE 3:** Haplotype analysis of the *CLEC16A* variants in PD patients and controls.

Haplotype^a^	Control, n (%)	PD, n (%)	*P*	OR (95% CI)
GAGAG	453.5 (44.99)	399.7 (38.81)	—	1.00
AGCGA	182.8 (18.13)	197.0 (19.13)	0.12	1.22 (0.95–1.56)
AAGAG	93.3 (9.26)	120.9 (11.74)	0.02^b^	1.47 (1.06–2.04)
AACGG	82.2 (8.15)	98.3 (9.54)	0.03^b^	1.47 (1.04–2.07)
GACAG	72.2 (7.16)	75.8 (7.38)	0.34	1.20 (0.82–1.74)
Total	1008.0 (100)	1030.0 (100)	0.034^b^	—

a Haplotype alleles were in the order of rs6498169, rs12708716, rs12917716, rs7200786, and rs2903692. Haplotypes with frequency <3% in both PD patients and controls were excluded from the analysis.

b *P* < 0.05. CI, confidence interval; OR, odds ratio; PD, Parkinson’s disease.

To characterize the highest-risk haplotype of *CLEC16A* towards PD, a backward elimination model was employed. Results showed that the strongest PD-associated haplotype was AAG in the order of rs6498169, rs12708716, and rs2903692 from the best 3-variant model (*p* = 0.0047, OR = 1.42, 95% CI = 1.11–1.82; Table 4).

**TABLE 4:** The highest-risk haplotype analysis of *CLEC16A* in association with PD.

Variants, n	Haplotype^a^	Control, n (%)	PD, n (%)	*P*	OR (95%CI)
5	AAGAG	93.3 (9.26)	120.9 (11.74)	0.02	1.47 (1.06–2.04)
4	AAG-G	96.7 (9.59)	129.4 (12.56)	0.011	1.54 (1.11–2.15)
3	AA--G	185.1 (18.36)	233.1 (22.63)	0.0047	1.42 (1.11–1.82)
2	A---G	218.0 (21.63)	264.8 (25.71)	0.017	1.32 (1.05–1.65)

a Haplotype alleles were in the order of rs6498169, rs12708716, rs12917716, rs7200786, and rs2903692. Haplotypes with frequency <3% in both PD patients and controls were excluded from the analysis. A hyphen indicates the eliminated variant at that position.

CI, confidence interval; OR, odds ratio; PD, Parkinson’s disease.

### Function Annotations of the PD-Associated CLEC16A Variants

Function annotations of the PD-associated variants were performed using the HaploReg, RegulomeDB, and GTEx. Based on the HaploRreg (Tables 5 and Supplementary Table S3), rs6498169 was located within enhancer histone marks in 13 tissues, DNase hypersensitivity regions in 7 tissues, and the region of GATA2 (GATA binding protein 2) binding site, and was predicted to significantly alter the binding motif of the HES1 (hes family bHLH transcription factor 1) transcription factor. Rs7200786 was located within enhancer histone marks in 13 tissues, and DNase hypersensitivity regions in 3 tissues and was predicted to significantly change the binding motifs of the CCNT2 (cyclin T2) and EBF (early B cell factor) transcription factors.

**TABLE 5:** Function annotations of the PD-associated *CLEC16A* variants.

Tools	rs6498169	rs7200786	
HaploReg			
Enhancer histone marks^a^	13 tissues	13 tissues	
DNase hypersensitivity^a^	7 tissues	3 tissues	
Proteins bound	GATA2	—	
Motifs changed	HES1	CCNT2; EBF	
RegulomeDB			
Score	1b	4	
GTEx (eQTL)			
Genes affected	*RMI2*	*CLEC16A*	*RMI2*
Tissues affected^b^	Cerebellum; cortex; frontal cortex; hypothalamus; nucleus accumbens; putamen; spinal cord	Amygdala; cerebellum; cerebellar hemisphere	Anterior cingulate; cerebellum; cortex; nucleus accumbens; putamen; spinal cord	
GTEx (sQTL)			
Genes affected	*CLEC16A*	*CLEC16A*	
Tissues affected^c^	Testis	Testis	

a Tissues were detailed in Supplementary Table S3.

b Brain tissues were listed herein. Full tissue lists were shown in Supplementary Figures S1–S3.

c Only testis data were available. Details were shown in Supplementary Figure S4.

eQTL, expression quantitative trait locus; PD, Parkinson’s disease; sQTL, splicing quantitative trait locus.

In RegulomeDB (Table 5), where the score <3 indicates a relatively high possibility of potential regulatory function (Luciano et al., 2018), rs6498169 was predicted with a score of 1b, representing that this variant is highly likely to affect transcription factor binding, certain motifs, DNase footprint and peaks, and potentially affect the expression of target genes. The score of rs7200786 was 4, representing that this variant may affect transcription factor binding and DNase peaks.

By using GTEx (Table 5; Supplementary Figures S1–S3), rs6498169 was suggested to be significant eQTL of the *RMI2* (RecQ mediated genome instability 2) expression in brain tissues such as cortex (*p* = 0.0015), cerebellum (*p* = 0.008) and nucleus accumbens (*p* = 0.008). Rs7200786 was as significant eQTL of the *RMI2* expression in brain tissues such as nucleus accumbens (*p* = 0.00071), cortex (*p* = 0.0028) and putamen (*p* = 0.0033), as well as the *CLEC16A* expression in brain tissues such as cerebellar hemisphere (*p* = 0.0012) and amygdala (*p* = 0.008). However, both rs6498169 and rs7200786 were not associated with the expression of *RMI2* and *CLEC16A* in the substantia nigra. Results of the sQTL analysis showed that the risk alleles of both rs6498169 and rs7200786 (A and G, respectively) were significantly associated with an increased intron-excision ratio, which potentially leads to elevation of certain variant expression of *CLEC16A* (Supplementary Figure S4).

## Discussion

Immunity disturbance is increasingly considered to be important in PD pathogenesis. The clinical observations of risk association between PD and autoimmune diseases indicate the possibility of having common genetic risk loci in between. By studying the five autoimmune disease associated *CLEC16A* variants in a Chinese cohort, we demonstrate that *CLEC16A* is pleiotropic for modulating PD risk. Two loci, rs6498169 and rs7200786, are recessively associated with PD susceptibility.

CLEC16A is involved in regulation of autophagy, T cell selection, antigen presentation, and neurodegeneration as suggested earlier (Soleimanpour et al., 2014; Schuster et al., 2015; van Luijn et al., 2015; Redmann et al., 2016). Autophagy plays a role, not only for substance clearance and recycling, but also in the presentation of antigenic peptides to the receptor of T cells in the context of antigen-presenting cells and major histocompatibility complex class II (Bonam and Muller, 2020). While disruption of the autophagy pathway serves as one of the key mechanisms in PD (Michel et al., 2016), PD patients are indeed observed with dysregulated innate and adaptive immune responses, particularly in those carrying autophagy-related gene mutations such as in Parkin and LRRK2 (leucine rich repeat kinase 2). Besides the previously mentioned α-synuclein reactive T cell responses (Sulzer et al., 2017; Lindestam Arlehamn et al., 2020), B cell-produced autoantibodies against α-synuclein antigen, GM1 gangliosides, and neuronal antigens are also shown to be elevated in blood and/or cerebral spinal fluid of PD patients (Zappia et al., 2002; van de Warrenburg et al., 2008; Scott et al., 2018). Hence, besides the identified genetic connection, *CLEC16A* may be functionally possible to participate in PD.

Our results show that rs6498169 and rs7200786 are associated with PD susceptibility. The modulation of rs7200786 on PD risk is consistent with a previous report in the Italian population (Strafella et al., 2021). The A of rs6498169 and the G of rs7200786 are recessive risk alleles. However, these two alleles are protective against multiple sclerosis and rheumatoid arthritis (Vitale et al., 2002; International Multiple Sclerosis Genetics et al., 2007; Martinez et al., 2010; Skinningsrud et al., 2010; International Multiple Sclerosis Genetics et al., 2013). When the relationship between PD and multiple sclerosis remains in debate (Li et al., 2012; Pedemonte et al., 2013; Nielsen et al., 2014), PD risk, indeed, appears to be negatively correlated with rheumatoid arthritis (Bacelis et al., 2021; Li et al., 2021). These results suggest that the risk variants of *CLEC16A* may have differential actual impact on PD and certain autoimmune diseases. Interestingly, a recent GWAS pooling study of European ancestry identified 17 shared susceptibility loci between autoimmune diseases and PD (Witoelar et al., 2017). Similar to our case, these loci have not been previously reported by PD GWAS studies (Obeso et al., 2017), but partially reported by individual polymorphism studies. Thus, a variety in research designs and ethnicities may still be valuable in searching for new genetic risks. Conversely, these findings may need further validation in additional populations. Being noted, the above European study identified none of the *CLEC16A* variants (Witoelar et al., 2017). To understand the discordance, we examined allele frequencies of the five variants in Europeans and East Asians in the gnomAD (https://gnomad.broadinstitute.org/). Interestingly, the two PD-associated *CLEC16A* variants happen to be with inverted major and minor allele distributions in these two ethnicities. In detail, the G allele frequency of rs6498169 is at 0.358 and 0.554, and the A allele frequency of rs7200786 is at 0.461 and 0.660, respectively, in Europeans and East Asians (Supplementary Table S4). In this case, the pleiotropic discordance may partially attribute to the ethnicity-associated evolutional divergency in *CLEC16A* genetic variation.

It is known that haplotypes are more powerful for the detection of susceptibility alleles than individual variants (Gabriel et al., 2002). By analyzing the five variants, we identified two haplotypes, AAGAG and AACGG (in the order of rs6498169, rs12708716, rs12917716, rs7200786, and rs2903692), serving as risk factor. Further analysis of the effect polymorphisms suggests that the AAG of rs6498169, rs12708716 and rs2903692 represents the core haplotype associated with PD. Results of the functional annotation analyses suggest that rs6498169 is highly probable to affect transcription factor binding and target gene expression, such as through the modulation of GATA2 and HES1 binding. In contrast, rs7200786 is with relatively less probability to be functional. These results appear to be in line with the above highest-risk haplotype analysis, which shows that the rs6498169 locus, but not rs7200786, is within the core in association with PD. Based on the quantitative trait locus analysis, the *CLEC16A* expression may be affected by rs6498169 in a splicing-regulating way and by rs7200786 in both expression- and splicing-regulating ways. In addition, rs6498169 and rs7200786 may also affect the *RMI2* expression in an expression-regulating way. RMI2 is a component of the BLM (Bloom syndrome RecQ like helicase) complex and is essential for genome stability (Hudson et al., 2016). However, the effects of these two variants on gene expression appear mainly in brain regions other than the substantia nigra. Indeed, although the substantia nigra is most profoundly affected in PD, other brain regions are also important for PD pathogenesis and clinical manifestations, such as putamen, cortex and amygdala (Kalia and Lang, 2015).

In conclusion, the present study demonstrates that the autoimmune disease associated *CLEC16A* genetic variants are associated with PD susceptibility in Han Chinese. Specifically, the A of rs6498169 and the G of rs7200786 serve as recessive risk alleles towards PD. These findings provide genetic insights into the pleiotropic role of *CLEC16A* and strengthen the link between PD and autoimmune diseases.

## Data Availability

The datasets presented in this study can be found in online repositories. The name of the repository and link can be found below: Dryad; https://datadryad.org/stash/share/_yc2e6e8jRgT3dCBI-WN2w4NZNY1CqRkqiSnD4q1h7w.
