# Supplementary material for: Autoimmune Disease Associated CLEC16A Variants Convey Risk of Parkinson’s Disease in Han Chinese
Source: Front Genet. 2022 Mar 30;13:856493. doi: 10.3389/fgene.2022.856493 (PMC9007333; doi:10.3389/fgene.2022.856493)
Supplement: Supplementary file 1 [file DataSheet1.docx]

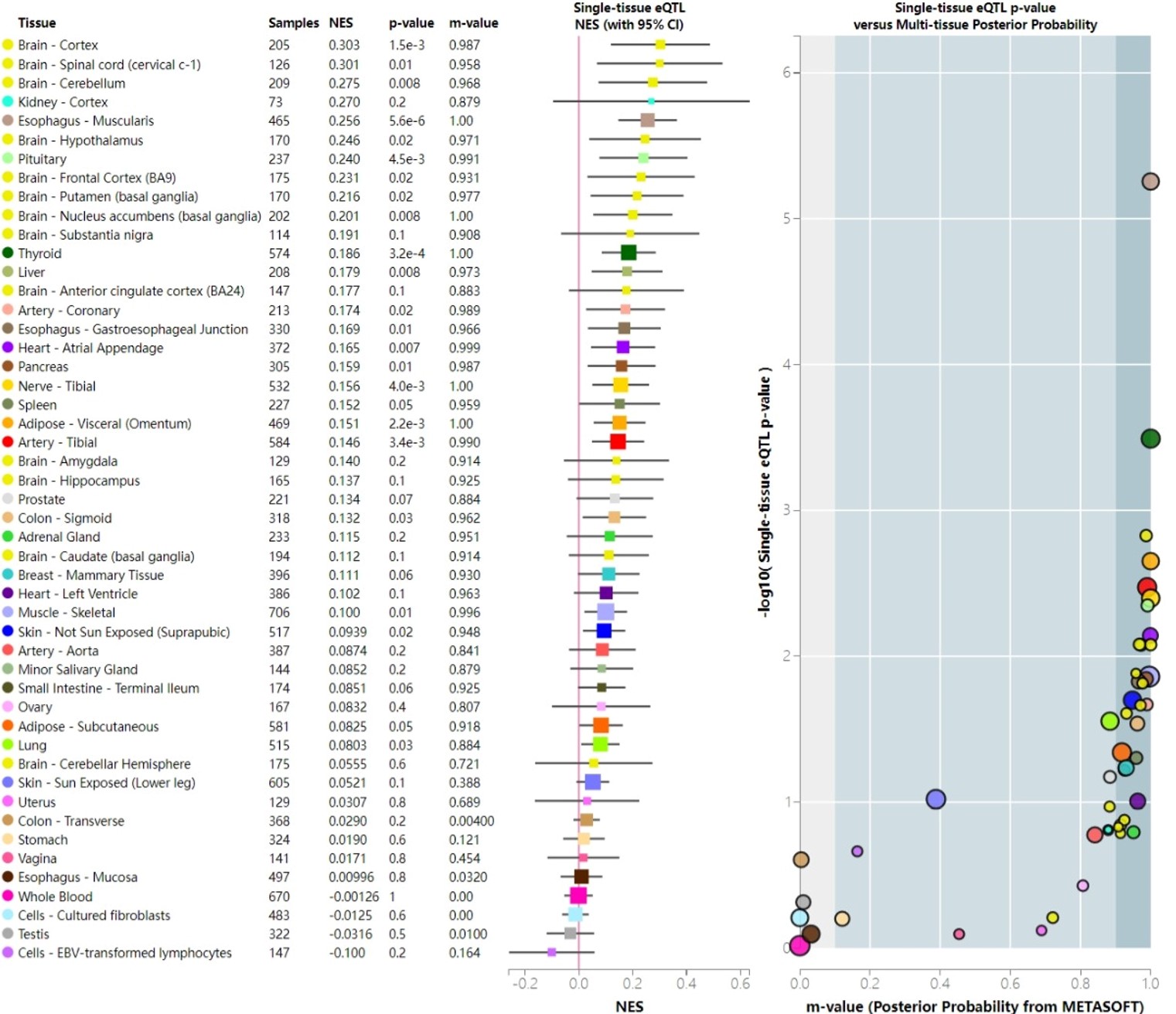


**Figure S1.** Multi-tissue eQTL correlation analysis between rs6498169 and the *RMI2* expression. The plot was generated from GTEx. Significance was considered at *P* < 0.05. eQTL, expression quantitative trait locus.


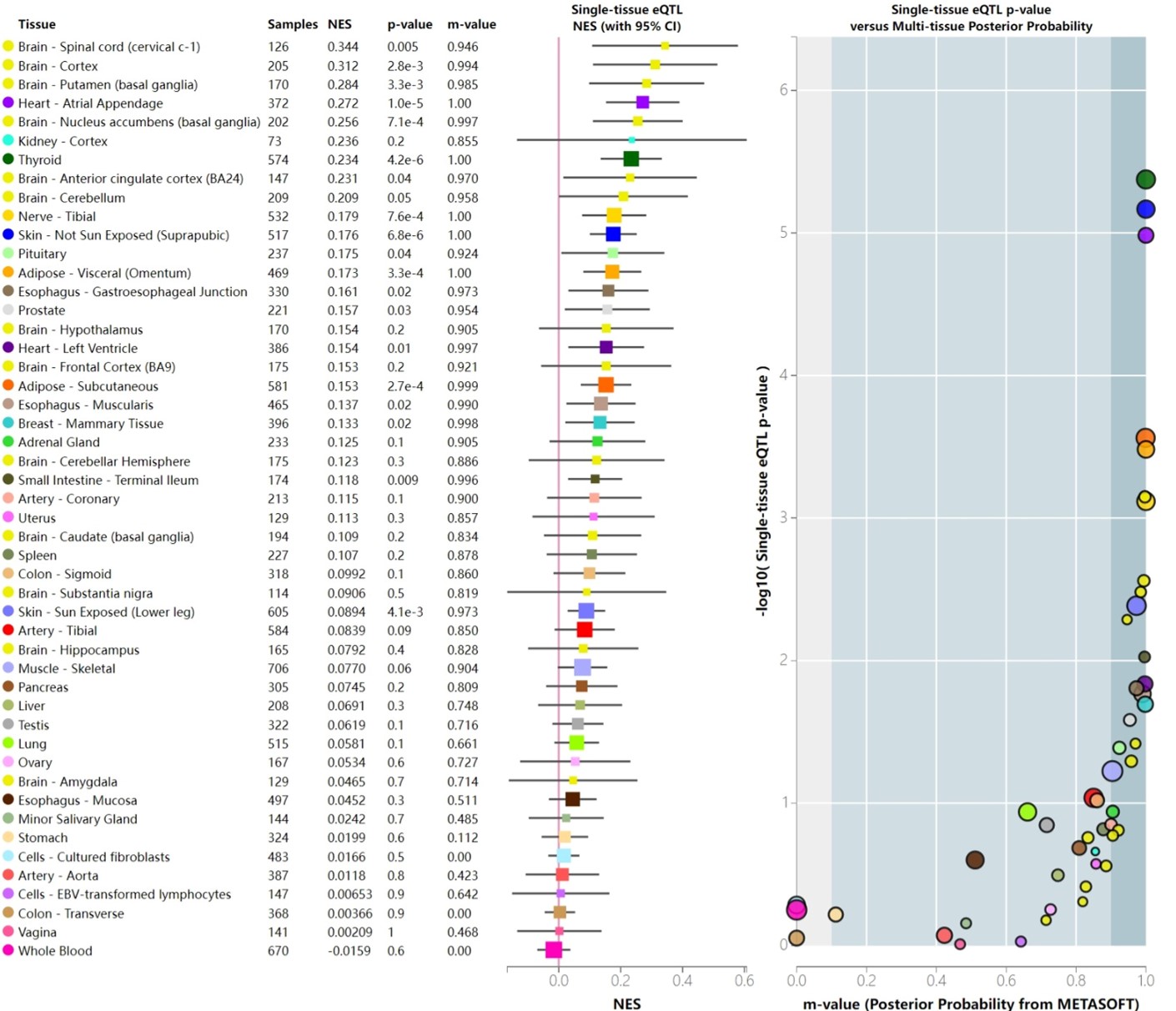


**Figure S2.** Multi-tissue eQTL correlation analysis between rs7200786 and the *RMI2* expression. The plot was generated from GTEx. Significance was considered at *P* < 0.05. eQTL, expression quantitative trait locus.


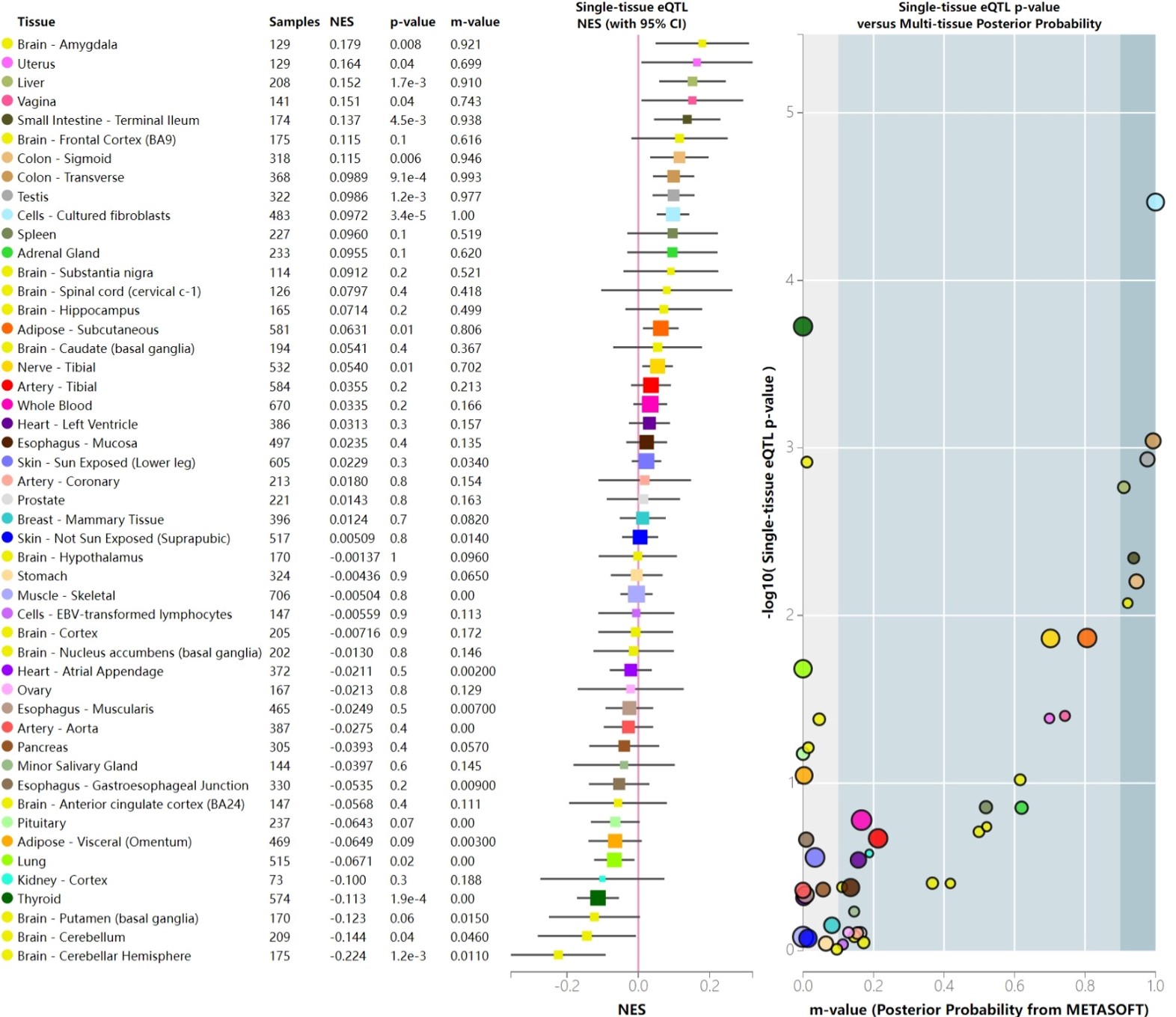


**Figure S3.** Multi-tissue eQTL correlation analysis between rs7200786 and the *CLEC16A* expression. The plot was generated from GTEx. Significance was considered at *P* < 0.05. eQTL, expression quantitative trait locus.


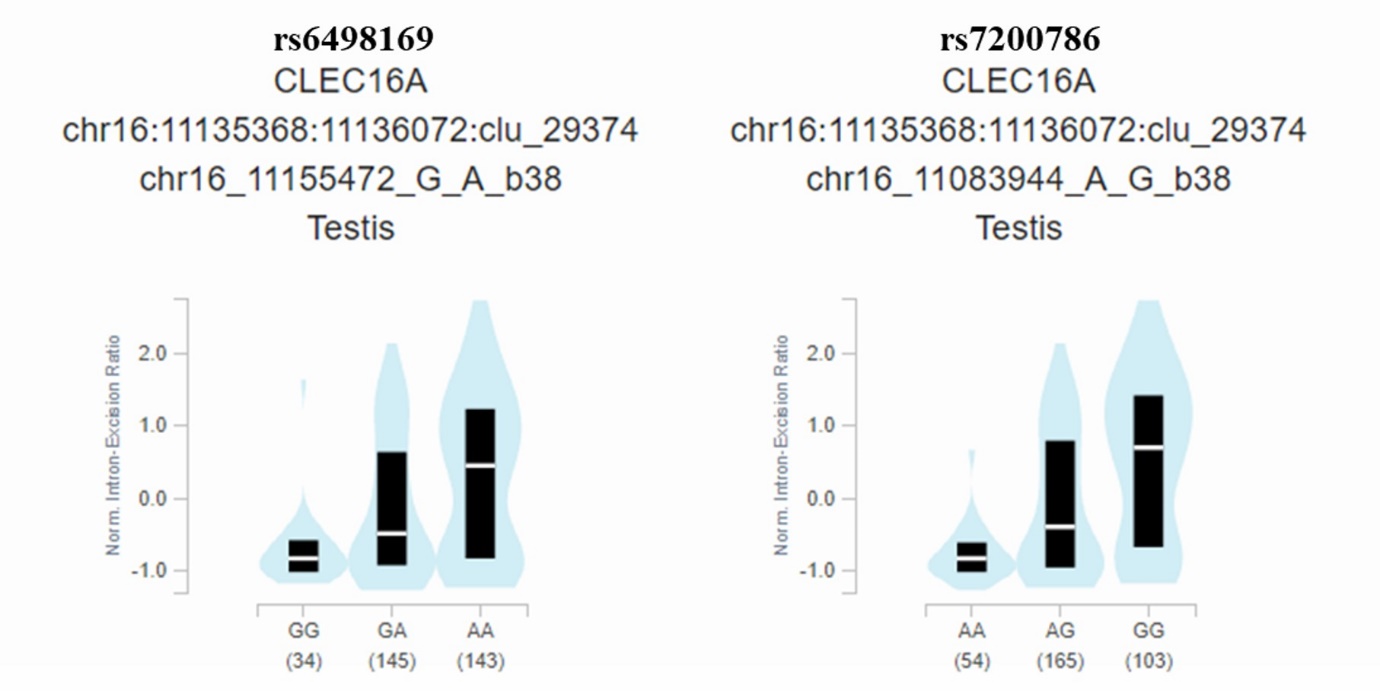


**Figure S4.** The sQTL data of rs6498169 and rs7200786 obtained from GTEx. *P* = 2.8 × 10^-8^ and 6.5 × 10^-16^, respectively for rs6498169 and rs7200786. Significance was considered at *P* < 0.05. sQTL, splicing quantitative trait locus.

**Table S1. Primers for SNaPshot and PCR**

| **Method** | **Variant** | **Primers (5’-3’)** | **Primers for single-base extension (5’-3’)** |
| --- | --- | --- | --- |
| SNaPshot |  |  |  |
|  | rs6498169 | Forward: TTGCTGCTCTTCAGGTTT | GGTTTCTCCCCTGCAGATAGCAGAAGGCTC |
|  |  | Reverse: GAGTCAGATAGAATCAGGGAG |  |
|  | rs12708716 | Forward: GGGGACAAACATCCAAAC | CTCTCGGGTCTTCAGCTAGTCCTCTGGGCAGTAGGGAGAATCCT |
|  |  | Reverse: CCTGGAAACAAGCCTAAC |  |
|  | rs12917716 | Forward: AAAAGTGGGCGGTAATGT | TTTAGAGCAAGAACCAGAGAGAAGAAAGAAAGGAAAGAGAAGATGAAAAAGAAAAAAG |
|  |  | Reverse: CTCTGTTCCTGGAGTGGT |  |
|  | rs7200786 | Forward: AAAGGAGCCCAGAATAAACA | GTGTTCTACCCGGAATGGAAACCAGCTTTTCACCTTGTCCAGGAGGTTGGG |
|  |  | Reverse: TTGGACTCTGCTGGTGGA |  |
| PCR/Sequencing |  |  | **Product** |
|  | rs2903692 | Forward: CTGTGCTAAGAACTGGGAAAT | 279 bp |
|  |  | Reverse: GGACAGGTAATCGGGAGC |  |

PCR, polymerase chain reaction.

**Table S2. Genotype and allele frequencies of five *CLEC16A* variants in patients of PD subtypes and controls**

| **Variant** | **Genotype, n (%)** | | | ***P*** | **Allele, n (%)** | | ***P*** | **OR (95% CI)** |
| --- | --- | --- | --- | --- | --- | --- | --- | --- |
| **rs6498169** | **GG** | **GA** | **AA** |  | **G** | **A** |  |  |
| Control | 151 (30.0) | 264 (52.4) | 89 (17.7) |  | 566 (56.2) | 442 (43.8) |  |  |
| PIGD | 25 (28.1) | 34 (38.2) | 30 (33.7) | 0.002* | 84 (47.2) | 94 (52.8) | 0.027 | 1.433 (1.041-1.972) |
| TD | 40 (25.6) | 75 (48.1) | 41 (26.3) | 0.058 | 155 (49.7) | 157 (50.3) | 0.045 | 1.297 (1.006-1.673) |
| indeterminate | 7 (35.0) | 7 (35.0) | 6 (30.0) | 0.234 | 21 (52.5) | 19 (47.5) | 0.648 | 1.159 (0.615-2.182) |
| **rs12708716** | **AA** | **AG** | **GG** |  | **A** | **G** |  |  |
| Control | 293 (58.1) | 186 (36.9) | 25 (5.0) |  | 772 (76.6) | 236 (23.4) |  |  |
| PIGD | 52 (58.4) | 27 (30.3) | 10 (11.2) | 0.051 | 131 (73.6) | 47 (26.4) | 0.388 | 1.174 (0.816-1.689) |
| TD | 81 (51.9) | 68 (43.6) | 7 (4.5) | 0.325 | 230 (73.7) | 82 (26.3) | 0.300 | 1.166 (0.872-1.561) |
| indeterminate | 11 (55.0) | 7 (35.0) | 2 (10.0) | 0.607 | 29 (72.5) | 11 (27.5) | 0.550 | 1.241 (0.611-2.522) |
| **rs12917716** | **GG** | **GC** | **CC** |  | **G** | **C** |  |  |
| Control | 173 (34.3) | 258 (51.2) | 73 (14.5) |  | 604 (59.9) | 404 (40.1) |  |  |
| PIGD | 33 (37.1) | 36 (40.4) | 20 (22.5) | 0.082 | 102 (57.3) | 76 (42.7) | 0.512 | 1.114 (0.807-1.538) |
| TD | 39 (25.0) | 86 (55.1) | 31 (19.9) | 0.056 | 164 (52.6) | 148 (47.4) | 0.021 | 1.349 (1.045-1.742) |
| indeterminate | 9 (45.0) | 7 (35.0) | 4 (20.0) | 0.363 | 25 (62.5) | 15 (37.5) | 0.744 | 0.897 (0.467-1.722) |
| **rs7200786** | **AA** | **AG** | **GG** |  | **A** | **G** |  |  |
| Control | 220 (43.7) | 239 (47.4) | 45 (8.9) |  | 679 (67.4) | 329 (32.6) |  |  |
| PIGD | 39 (43.8) | 33 (37.1) | 17 (19.1) | 0.010 | 111 (62.4) | 67 (37.6) | 0.192 | 1.246 (0.895-1.734) |
| TD | 59 (37.8) | 72 (46.2) | 25 (16.0) | 0.036 | 190 (60.9) | 122 (39.1) | 0.035 | 1.325 (1.019-1.723) |
| indeterminate | 10 (50.0) | 6 (30.0) | 4 (20.0) | 0.139 | 26 (65.0) | 14 (35.0) | 0.755 | 1.111 (0.573-2.156) |
| **rs2903692** | **GG** | **GA** | **AA** |  | **G** | **A** |  |  |
| Control | 289 (57.3) | 188 (37.3) | 27 (5.4) |  | 766 (76.0) | 242 (24.0) |  |  |
| PIGD | 47 (52.8) | 37 (41.6) | 5 (5.6) | 0.723 | 131 (73.6) | 47 (26.4) | 0.492 | 1.136 (0.790-1.633) |
| TD | 79 (50.6) | 70 (44.9) | 7 (4.5) | 0.237 | 228 (73.1) | 84 (26.9) | 0.297 | 1.166 (0.874-1.557) |
| indeterminate | 13 (65.0) | 5 (25.0) | 2 (10.0) | 0.420 | 31 (77.5) | 9 (22.5) | 0.827 | 0.919 (0.431-1.957) |

*, *P* < 0.01. CI, confidence interval; OR, odds ratio; PD, Parkinson’s disease; PIGD, postural instability/gait difficulty; TD, tremor dominant.

**Table S3. HaploReg-generated tissue lists for rs6498169 and rs7200786**

|  | **rs6498169** | **rs7200786** |  |
| --- | --- | --- | --- |
| Enhancer histone marks | H1 BMP4 Derived Trophoblast Cultured Cells, hESC Derived CD56+ Mesoderm Cultured Cells, IMR90 fetal lung fibroblasts Cell Line, Breast variant Human Mammary Epithelial Cells (vHMEC), Muscle Satellite Cultured Cells, Foreskin Fibroblast Primary Cells skin01, Foreskin Fibroblast Primary Cells skin02, Foreskin Keratinocyte Primary Cells skin02, Foreskin Keratinocyte Primary Cells skin03, Fetal Adrenal Gland, Left Ventricle, Placenta Amnion, Right Ventricle, Skeletal Muscle Male, Spleen, HeLa-S3 Cervical Carcinoma Cell Line, K562 Leukemia Cells, NH-A Astrocytes Primary Cells, NHEK-Epidermal Keratinocyte Primary Cells, NHLF Lung Fibroblast Primary Cells, Osteoblast Primary Cells | H1 BMP4 Derived Trophoblast Cultured Cells, H1 Derived Mesenchymal Stem Cells, IMR90 fetal lung fibroblasts Cell Line, Adipose Derived Mesenchymal Stem Cell Cultured Cells, Mesenchymal Stem Cell Derived Chondrocyte Cultured Cells, Muscle Satellite Cultured Cells, Foreskin Fibroblast Primary Cells skin01, Foreskin Fibroblast Primary Cells skin02, Foreskin Melanocyte Primary Cells skin03, Colon Smooth Muscle, Fetal Adrenal Gland, Fetal Lung, Placenta, Fetal Stomach, Fetal Thymus, Psoas Muscle, Stomach Smooth Muscle, Thymus, Dnd41 T Cell Leukemia Cell Line, HSMM Skeletal Muscle Myoblasts Cells, NH-A Astrocytes Primary Cells, NHDF-Ad Adult Dermal Fibroblast Primary Cells, NHLF Lung Fibroblast Primary Cells, Osteoblast Primary Cells | |
| DNase hypersensitivity | H1 BMP4 Derived Trophoblast Cultured Cells, H1 Derived Mesenchymal Stem Cells, IMR90 fetal lung fibroblasts Cell Line, HeLa-S3 Cervical Carcinoma Cell Line, K562 Leukemia Cells, NH-A Astrocytes Primary Cells, NHLF Lung Fibroblast Primary Cells | H1 Derived Neuronal Progenitor Cultured Cells, Primary T Cells from Peripheral Blood, HepG2 Hepatocellular Carcinoma Cell Line | |

**Table S4. Allele frequencies of five *CLEC16A* variants in Europeans and East Asians**

| **Variant** | **European** | |  | **East Asian** | |
| --- | --- | --- | --- | --- | --- |
|  | Reference allele | Alternative allele |  | Reference allele | Alternative allele |
| rs6498169 | G=0.358 | A=0.642 |  | G=0.554 | A=0.446 |
| rs12708716 | A=0.640 | G=0.360 |  | A=0.783 | G=0.217 |
| rs12917716 | G=0.567 | C=0.433 |  | G=0.590 | C=0.410 |
| rs7200786 | A=0.461 | G=0.539 |  | A=0.660 | G=0.340 |
| rs2903692 | G=0.655 | A=0.345 |  | G=0.779 | A=0.221 |
